# Trends and disparities in prevalence of cardiometabolic diseases by food security status in the United States

**DOI:** 10.1186/s12937-023-00910-4

**Published:** 2024-01-03

**Authors:** Junxiu Liu, Stella S. Yi, Rienna G. Russo, Carol R. Horowitz, Donglan Zhang, Janani Rajbhandari-Thapa, Dejun Su, Lu Shi, Yan Li

**Affiliations:** 1 Department of Population Health Science and Policy, Icahn School of Medicine at Mount Sinai, One Gustave L. Levy Place, Box 1077, New York, NY 10029 USA; 2 Department of Population Health, NYU Grossman School of Medicine, New York, NY USA; 3 Department of Medicine, Icahn School of Medicine at Mount Sinai, New York, NY USA; 4 Department of Foundations of Medicine, NYU Grossman Long Island School of Medicine, Mineola, NY USA; 5 Department of Health Promotion, College of Public Health, University of Nebraska Medical Center, Omaha, NE USA; 6 Department of Public Health Science, College of Behavioral, Social and Health Science, Clemson University, Clemson, SC USA; 7 School of Public Health, Shanghai Jiao Tong University School of Medicine, 227 S. Chongqing Rd, Shanghai, 200025 China; 8 Department of Health Policy and Management, College of Public Health, University of Georgia, Athens, GA USA

## Abstract

**Background:**

Previous studies have demonstrated the association between food security and cardiometabolic diseases (CMDs), yet none have investigated trends in prevalence of CMDs by food security status in the United States (US).

**Methods:**

Serial cross-sectional analysis of the US nationally representative data from National Health and Nutrition Examination Survey (1999–2018) was conducted among adults aged 20 years or older. Food security status was defined by the US Household Food Security Survey Module (full, marginal, low, and very low food security). We estimated the age-adjusted prevalence of CMDs including obesity, hypertension, diabetes, and coronary heart disease by food security status. Racial and ethnic disparities in age-adjusted prevalence of CMDs by food security status were also assessed.

**Results:**

A total of 49,738 participants were included in this analysis (weighted mean age 47.3 years; 51.3% women). From 1999 to 2018, the age-adjusted prevalence of CMDs was lower in full food secure group as compared with other groups. For example, trends in hypertension decreased from 49.7% (47.5-51.8%) to 45.9% (43.8-48.0%) (*P*-trend = 0.002) among the full and from 54.2% (49.9-58.5%) to 49.7% (46.8-52.6%) (*P*-trend = 0.02) among the marginal but remained stable among the low at 49.7% (47.9-51.6%) and among the very low at 51.1% (48.9-53.3%) (*P*-interaction = 0.02). Prevalence of diabetes increased from 8.85% (8.15-9.60%) to 12.2% (11.1-13.5%) among the full (*P*-trend < 0.001), from 16.5% (13.2-20.4%) to 20.9% (18.6-23.5%) (*P*-trend = 0.045) among the marginal and from 14.6% (11.1-19.0%) to 20.9% (18.8-23.3%) (*P*-trend = 0.001) among the low but remained stable at 18.8% (17.0-20.9) among the very low (*P*-trend = 0.35) (*P*-interaction = 0.03). Racial and ethnic differences in prevalence of CMD by food security status were observed. For example, among individuals with full food secure status, the prevalence of diabetes was 9.08% (95% CI, 8.60-9.59%) for non-Hispanic whites, 17.3% (95% CI, 16.4-18.2%) for non-Hispanic blacks, 16.1% (95% CI, 15.0-17.4%) for Hispanics and 14.9% (95% CI, 13.3-16.7%) for others.

**Conclusions and relevance:**

Prevalence of CMDs was greatest among those experiencing food insecurity, and food insecurity disproportionately affected racial/ethnic minorities. Disparities in CMD prevalence by food security status persisted or worsened, especially among racial/ethnic minorities.

## Introduction

Food insecurity, defined as limited or uncertain access to adequate food, is a key social determinant of health for both children and adults [1, 2]. When left unaddressed, food insecurity adversely affects cardiometabolic health, leading to multiple cardiometabolic diseases (CMDs) such as hypertension and type 2 diabetes, [3–5] and hampering efforts for disease management [6, 7]. A recent study demonstrated that an increase in food insecurity was associated with an increase in cardiovascular mortality at the county level [8]. In 2018, food insecurity affected 11.2% of U.S. households, or almost 37.2 million people [9]. It also disproportionately affects racial and ethnic minorities, as non-Hispanic Blacks and Hispanics were more than twice more likely to being food insecure compared to non-Hispanic Whites [9–12]. Since the beginning of the COVID-19 pandemic, food insecurity has increased tremendously across the U.S., potentially worsening racial and ethnic disparities [13–16].

Food insecurity exhibits a similar concern with the alarming increase of population-level cardiometabolic health issues, such as obesity, hypertension, and diabetes, over the past two decades [17–19]. We thus postulate that the worsening cardiometabolic health may be partially driven by the worsening cardiometabolic health outcomes among people who are most food insecure. To explore this hypothesis, we used a nationally representative data to examine the trends of major CMDs (i.e., obesity, hypertension, diabetes, coronary heart disease) by food security status over the past two decades. We also assessed the association of food insecurity with major CMDs, accounting for potential sociodemographic confounders. Finally, we assessed racial and ethnic disparities in the trends of CMDs by food security status.

## Methods

### Study design and population

The National Health and Nutrition Examination Survey (NHANES) is a series of cross-sectional, nationally representative surveys, administered by the Centers for Disease Control and Prevention’s National Center for Health Statistics (NCHS). NHANES samples the civilian noninstitutionalized US population residing in the 50 states and the District of Columbia using a complex, multistage probability survey design. Information is collected through standardized questionnaires during in-home interviews and physical examinations performed in the Mobile Examination Center (MEC). Since 1999, data have been released continuously in 2-year cycles. The survey protocol was approved by NCHS Research Ethics Review Board, and all participants provided written informed consent. Given that NHANES data are publicly available and deidentified, this analysis was exempt from institutional review board.

This study was conducted across 10 cycles of NHANES from 1999 to 2000 through 2017–2018. The analytical sample included men and nonpregnant women 20 years and older who completed the food security questionnaire and physical examination (N = 49,738).

### Assessment of food security status

Information on food security status was collected through the US Household Food Security Survey Module developed by the US Department of Agriculture [20]. The module consists of 18 items, including 10 items for adults in the household and 8 for children. These items capture the experience, perception, and anxiety that a person may have related to food insecurity such as having insufficient food budget, feeling that food is inadequate in quality or quantity, and experiencing the physical sensation of hunger and weight loss.

We used responses to the 10 items for adults for this study. Based on the number of affirmative responses to these items, the NCHS created four response levels, including full food security (0 affirmative responses), marginal food security (1–2 affirmative responses), low food security (3–5 affirmative responses), and very low food security (6–10 affirmative responses). We used these four categories of food security level in our analyses.

### Assessment of health outcomes

CMD outcomes in this analysis included obesity, hypertension, diabetes, and coronary heart disease (CHD). Body mass index for obesity, blood pressure measures and blood samples were collected in the MEC by trained health technicians and phlebotomists according to standardized protocols. Obesity was defined as body mass index (BMI) ≥ 30 kg/m^2^. Hypertension was defined as having at least one of the following conditions: systolic blood pressure (BP) ≥ 130 mmHg, diastolic BP ≥ 80, or currently taking BP medications. Diabetes was defined as self-reported prior diagnosis, fasting plasma glucose ≥ 126 mg/dL, or HbA1c ≥ 6.5%. CHD was defined as prior diagnosis of myocardial infarction, angina, or any other type of CHD.

### Assessment of sociodemographic characteristics

Sociodemographic characteristics including age, sex, race/ethnicity, educational attainment, and household income level were self-reported during in-home interviews. Race/ethnicity was defined as non-Hispanic White, non-Hispanic Black, Hispanic, and other, according to the categories provided by the NCHS. Educational attainment was defined as the highest grade or level of school completed or the highest degree received and categorized into four groups (less than high school, high school graduate/GED or equivalent, some college, or college graduate or above). Household income levels were defined by the family income to poverty ratio (IPR) and grouped into levels (low-income, IPR < = 1.0; middle-income IPR > 1 and < 4; and high-income, IPR ≥ 4.0) based on the thresholds used in the Patient Protection Affordable Care Act, in which adults with a IPR between 1 and 4 are eligible for insurance subsidies.

### Statistical analysis

Survey analysis procedures were applied in all analyses accounting for sample weights, stratification, and clustering of the complex survey design to ensure nationally representative estimates. Due to limited sample size, trends in CMDs by food security status were presented for every two combined cycles (1999–2002, 2003–2006, 2007–2010, 2011–2014, 2015–2018).

The estimated prevalence of CMD outcomes (obesity, hypertension, diabetes, and CHD) were estimated by NHANES cycles. Those estimates were age adjusted to the 2010 US census population using standardizing proportions for the age groups 22–44 years, 45–64 years, and 65 years or older, allowing comparisons independent of age. Survey-weighted logistic regression was used to calculate a *P*-value for trend across cycles. To evaluate potential differences in trends by food security status, a survey-weighted Wald F statistic was applied to test for an interaction between food security status and the combined two 2-year survey cycle.

Multivariable logistic regression models were used to investigate the relationship between food security status and the prevalence of obesity, diabetes, CHD, and hypertension. Co-variates included in the model were age, sex, race/ethnicity, educational attainment, and income. Multivariable adjusted odds ratios (aORs) and corresponding 95% confidence intervals (CIs) are presented. To assessed racial and ethnic disparities in the trends of CMDs by food security status, we conducted subgroup analysis that stratified race/ethnicity.

All statistical analyses were performed using Stata version 14.0 (Stata Corp, College Station). A two-sided *P* values < 0.05 was considered statistically significant. *P*-values were not adjusted for multiple testing and should be interpreted as exploratory.

## Results

### Participant characteristics

From 1999 to 2018, a total of 49,738 nonpregnant participants aged 20 years and older were included in the NHANES. Table 1 presents sociodemographic characteristics of the sample by food security status. The percentages of adults reported 79.5% (95% CI, 78.6-80.5%) living in households with full food security, 7.67% (7.21-8.15%) in households with marginal food security, 7.99% (7.53-8.48%) in households with low food security, and 4.78% (4.43-5.15%) in households with very low food security. Young adults, non-Hispanic Black and Hispanic individuals, and people with low educational attainment and low-income family households were more likely to report having low/very low food security. Individuals who were married or lived with partner, never smoked, had insurance coverage and non-SNAP participants were more likely to report having full food security status.

**Table 1:** Characteristics of study population by food security status, NHANES 1999–2018

Characteristics, N (%)	Food security status, No. (Survey weighted %) ^a^			
	Full (n = 35,599)	Marginal (n = 5,113)	Low (n = 5,734)	Very low (n = 3,292)
**Age group**				
20–44	13,442 (43.1)	2,517 (57.8)	2,945 (60.4)	1,632 (57.9)
45–64	11,728 (36.3)	1,674 (30.5)	1,914 (30.6)	1,190 (33.3)
≥ 65	10,429 (20.6)	922 (11.7)	875 (9.01)	470 (8.77)
**Sex**				
Men	17,903 (49.2)	2,379 (45.7)	2,722 (47.2)	1,600 (47.3)
Women	17,696 (50.8)	2,734 (54.3)	3,012 (52.8)	1,692 (52.6)
**Race/ethnicity**				
Non-Hispanic white	17,862 (74.1)	1,453 (47.7)	1,511 (44.5)	1,155 (51.7)
Non-Hispanic black	6,994 (9.26)	1,347 (18.5)	1,346 (17.4)	881 (18.9)
Hispanic	7,241 (9.78)	1,831 (25.8)	2,460 (31.6)	1,064 (22.9)
Others	3,502 (6.84)	482 (7.98)	417 (6.51)	192 (6.55)
**Education**				
<High school	7,821 (13.6)	1,839 (27.1)	2,580 (35.8)	1,332 (32.2)
High school or GED	8,001(22.9)	1,308 (29.5)	1,380 (27.3)	848 (29.3)
Some college	10,227 (30.7)	1,433 (31.4)	1,386 (28.8)	920 (31.9)
College graduate or above	9,503 (32.8)	522 (12.0)	381 (8.10)	184 (6.59)
Missing	47 (0.08)	11 (0.19)	7 (0.16)	8 (0.18)
**Family income (IPR)**				
≤ 1.0	4,102 (7.84)	1,588 (26.7)	2,350 (35.7)	1,581 (44.1)
1.0–4.0	17,390 (44.0)	2,870, 59.5	2,748 (53.0)	1,474 (48.9)
≥ 4.0	11,315 (41.8)	278 (7.44)	154 (3.85)	42 (2.63)
Missing	2,792 (6.20)	377 (6.35)	482 (7.45)	195 (4.31)
Marital status				
Married or living with partner	21,931 (65.3)	2,808 (55.7)	3,109 (54.2)	1,460 (44.9)
Separated/divorced/widowed	7,685 (17.4)	1,227 (22.2)	1,375 (22.2)	1,039 (29.3)
Not married	5,598 (16.1)	1,050 (21.5)	1,213 (23.0)	780 (25.5)
Missing	385 (1.23)	28 (0.59)	37 (0.57)	13 (0.35)
Smoking status				
Never	19,806 (55.5)	2,699 (50.4)	2,883 (47.6)	1,409 (39.6)
Current	6,196 (18.1)	1,342 (29.2)	1,694 (33.4)	1,304 (43.9)
Former	9,566 (26.3)	1,069 (20.4)	1,148 (18.9)	578 (16.4)
Missing	31 (0.05)	3 (0.04)	9 (0.18)	1 (0.01)
Insurance coverage				
Yes	30,194 (87.0)	3,484 (68.9)	3,614 (63.3)	2,054 (63.4)
No	5,405 (13.0)	1,629 (31.1)	2,120 (36.7)	1,238 (36.6)
SNAP participation				
Yes	2,601 (4,93)	1,111 (20.0)	1,533 (25.7)	1,079 (31.6)
No	32,998 (95.1)	4,002 (80.0)	4,201 (74.3)	2,213 (68.4)

Abbreviation: NHANES = National Health and Nutrition Examination Survey; SNAP, Supplemental Nutrition Assistance Program. CI, Confidence Interval. GED = general equivalency diploma

^a^ Data were weighted to be nationally representative

### Trends in the prevalence of CMDs by food security status

**Fig. 1:**
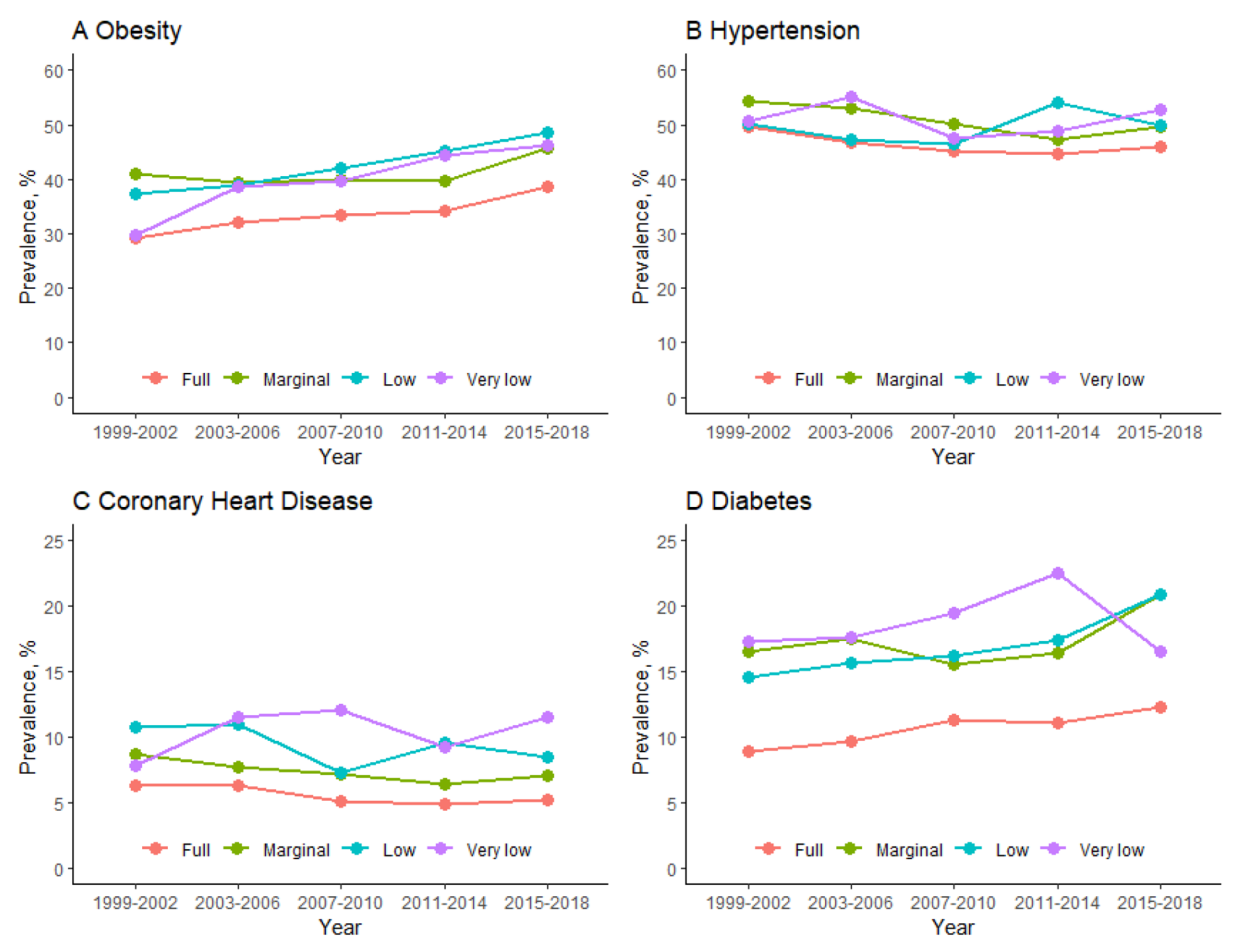
Trends in age-adjusted prevalence of obesity, hypertension, coronary heart disease, and diabetes by food security status among US adults aged 20 years or older by NHANES cycle from 1999 to 2018. The *P* values for trend in prevalence of obesity are < 0.001 for the full, 0.03 for the marginal, < 0.001 in the low, and 0.004 for very low and the *P* value for their interaction is 0.25. The *P* values for trend in prevalence of hypertension are 0.002 for the full, 0.02 for the marginal, 0.22 in the low, and 0.49 for very low and the *P* value for their interaction is 0.02. The *P* values for trend in prevalence of coronary heart disease are 0.02 for the full, 0.42 for the marginal, 0.19 in the low, and 0.67 for very low and the *P* value for their interaction is 0.66. The *P* values for trend in prevalence of diabetes are < 0.001 for the full, 0.045 for the marginal, 0.001 in the low, and 0.35 for very low and the *P* value for their interaction is 0.03

Figure 1 presents the prevalence of four major CMDs by food security status from 1999 to 2018. The age-adjusted prevalence of obesity increased among the full from 29.2% (95% CI, 27.5-31.1%) to 38.7% (35.8-41.5%) (P-trend < 0.001), marginal from 40.9% (95% CI, 35.3-46.8%) to 45.6% (95% CI, 42.1-49.3%) (P-trend = 0.03), low from 37.4% (32.7-42.3%) to 48.5% (44.5-52.5%) (P-trend < 0.001), and very low food security from 29.8% (22.6-38.1%) to 46.1% (40.9-51.4%) (P-trend = 0.004) groups (P-interaction = 0.25). For hypertension, the age-adjusted prevalence of hypertension decreased among the full from 49.7% (47.5-51.8%) to 45.9% (43.8-48.0%) (P-trend = 0.002) and marginal from 54.2% (49.9-58.5%) to 49.7% (46.8-52.6%) (P-trend = 0.02) food security groups but remained stable for the low [50.2% (45.2-55.1%) to 49.9% (45.8-54.0%), P-trend = 0.22] and very low [50.7% (44.1-57.2%) to 52.9% (49.1-56.6%), P-trend = 0.49] food security groups (P-interaction = 0.02).

Over the study period, the age-adjusted diabetes prevalence increased among the full from 8.85% (8.15-9.60%) to 12.2% (11.1-13.5%) (P-trend < 0.001), marginal from 16.5% (13.2-20.4%) to 20.9% (18.6-23.5%) (P-trend = 0.045) and low from 14.6% (11.1-19.0%) to 20.9% (18.8-23.3%) (P-trend = 0.001) food security groups but remained stable among the very low [17.3% (13.1-22.4%) to 16.5% (13.2-20.5%), P-trend = 0.35] food security group (P-interaction = 0.03). The age-adjusted CHD prevalence decreased among the full from 6.33% (5.59-7.16%) to 4.81% (3.97-5.83%) (P-trend = 0.02) food security group but remained stable among the marginal [7.16% (3.28-14.9%) to 6.61% (4.78-9.07%), P-trend = 0.42], low [10.5% (5.86-18.0%) to 8.41% (6.26-11.2%), P-trend = 0.19] and very low [8.26% (3.80-17.0%) to 8.94% (5.63-13.9%), P-trend = 0.67] food security groups (P-interaction = 0.66).

### Association of food security status with CMD

In multivariable logistic regression models, food security status was found to be significantly associated with the prevalence of obesity, diabetes, and CHD, but no association was found with the prevalence of hypertension (Table 2). Compared to the full food security group, the aORs (95% CIs) for obesity were 1.19 (1.09–1.30) for the marginal, 1.27 (1.16–1.38) for low, and 1.23 (1.09–1.39) for the very low food security groups. Compared to the full food security group, the aORs (95% CIs) for diabetes were 1.36 (1.21–1.54) for the marginal, 1.27 (1.12–1.44) for the low, and 1.49 (1.28–1.75) for the very low food security groups. Compared to the full food security group, the aORs (95% CIs) for CHD were 1.28 (1.09–1.50) for the marginal, 1.71 (1.41–2.06) for the low, and 1.88 (1.48–2.39) for the very low food security groups.

**Table 2:** Multivariable analysis of the association of food security status with obesity, hypertension, diabetes, and coronary heart disease, NHANES 1999–2018 (Data was weighted to be nationally representative)

Characteristics	Obesity		Hypertension		Diabetes		Coronary Heart Disease	
	Adjusted odds ratio (95% CI)	*P*-value	Adjusted odds ratio (95% CI)	*P*-value	Adjusted odds ratio (95% CI)	*P*-value	Adjusted odds ratio (95% CI)	*P*-value
**Food security**								
Full food secure	1.00 (reference)		1.00 (reference)		1.00 (reference)		1.00 (reference)	
Marginal food secure	1.19 (1.09–1.30)	< 0.001	1.11 (1.01–1.21)	0.05	1.36 (1.21–1.54)	< 0.001	1.28 (1.09–1.50)	0.01
Low food secure	1.27 (1.16–1.38)	< 0.001	1.07 (0.97–1.19)	0.20	1.27 (1.12–1.44)	0.001	1.71 (1.41–2.06)	< 0.001
Very low food secure	1.23 (1.09–1.39)	0.002	1.13 (1.0-1.29)	0.16	1.49 (1.28–1.75)	< 0.001	1.88 (1.48–2.39)	< 0.001
**Age category (y)**								
20–44	1.00 (reference)		1.00 (reference)		1.00 (reference)		1.00 (reference)	
45–64	1.28(1.20–1.37)	< 0.001	3.90 (3.62–4.20)	< 0.001	4.70 (4.28–517)	< 0.001	6.17 (5.08–7.50)	< 0.001
≥65	0.91 (0.84–0.98)	0.02	9.50 (8.64–10.5)	< 0.001	8.03 (7.18–8.98)	< 0.001	20.6 (17.1–24.8)	< 0.001
**Sex**								
Men	1.00 (reference)		1.00 (reference)		1.00 (reference)		1.00 (reference)	
Women	1.10 (1.04–1.17)	< 0.001	0.64 (0.61–0.68)	< 0.001	0.75 (0.70–0.82)	< 0.001	0.52 (0.46–0.58)	< 0.001
**Race/ethnicity**								
Non-Hispanic White	1.00 (reference)		1.00 (reference)		1.00 (reference)		1.00 (reference)	
Non-Hispanic Black	1.52 (1.42–1.63)	< 0.001	1.84 (1.71–1.98)	< 0.001	1.86 (1.72-2.0)	< 0.001	0.73 (0.65–0.82)	< 0.001
Hispanic	1.12 (1.04–1.21)	< 0.001	0.80 (0.74–0.87)	< 0.001	1.57 (1.43–1.73)	< 0.001	0.58 (0.51–0.66)	< 0.001
Others	0.56 (0.49–0.63)	< 0.001	1.11 (1.0-1.24)	0.05	1.74 (1.52-2.0)	< 0.001	0.95 (0.75–1.20)	0.29
**Education**								
<High school	1.00 (reference)		1.00 (reference)		1.00 (reference)		1.00 (reference)	
High school graduate or GED	1.12 (1.03–1.21)	0.004	0.96 (0.88–1.05)	0.36	0.80 (0.72–0.88)	< 0.001	0.83 (0.73–0.95)	0.009
Some college	1.12 (1.04–1.21)	< 0.001	0.87 (0.80–0.94)	0.001	0.81 (0.74–0.88)	< 0.001	0.82 (0.73–0.93)	0.001
College graduate or above	0.69 (0.62–0.76)	< 0.001	0.63 (0.58–0.70)	< 0.001	0.56 (0.50–0.63)	< 0.001	0.62 (0.53–0.72)	< 0.001
**Family income (IPR)**								
≤ 1.0	1.00 (reference)		1.00 (reference)		1.00 (reference)		1.00 (reference)	
1.0–4.0	1.13 (1.04–1.23)	0.003	1.08 (1.0-1.17)	0.06	0.90 (0.81–0.99)	0.04	0.84 (0.72–0.97)	< 0.001
≥4.0	1.04 (0.90–1.11)	0.49	1.01 (0.85–1.13)	0.92	0.72 (0.62–0.82)	< 0.001	0.66 (0.54–0.79)	< 0.001
Marital status								
Married	1.00 (reference)		1.00 (reference)		1.00 (reference)		1.00 (reference)	
Separated/divorced/widowed	0.99 (0.92–1.06)	0.71	1.21 (1.13–1.30)	< 0.001	1.04 (0.94–1.14)	0.45	1.22 (1.09–1.36)	< 0.001
No	0.82 (0.76–0.88)	< 0.001	0.80 (0.74–0.87)	< 0.001	0.86 (0.75–0.98)	0.02	0.73 (0.60–0.89)	0.002
Smoking status								
Never	1.00 (reference)		1.00 (reference)		1.00 (reference)		1.00 (reference)	
Current	0.69 (0.64–0.74)	< 0.001	0.85 (0.79–0.91)	< 0.001	0.82 (0.74–0.90)	< 0.001	1.49 (1.30–1.70)	< 0.001
Former	1.12 (1.05–1.20)	0.001	1.07 (1.0-1.15)	0.06	1.18 (1.09–1.29)	< 0.001	1.51 (1.35–1.68)	< 0.001
Insurance coverage								
Yes	1.00 (reference)		1.00 (reference)		1.00 (reference)		1.00 (reference)	
No	0.79 (0.74–0.85)	< 0.001	0.79 (0.74–0.85)	< 0.001	0.71 (0.64–0.78)	< 0.001	0.68 (0.57–0.81)	< 0.001
SNAP participation								
Yes	1.00 (reference)		1.00 (reference)		1.00 (reference)		1.00 (reference)	
No	0.73 (0.65–0.81)	< 0.001	0.91 (0.83, 1.0)	0.06	0.82 (0.74–0.92)	0.001	0.74 (0.63–0.88)	0.001

### Disparities in prevalence of CMDs by food security status across race/ethnicity

**Fig. 2:**
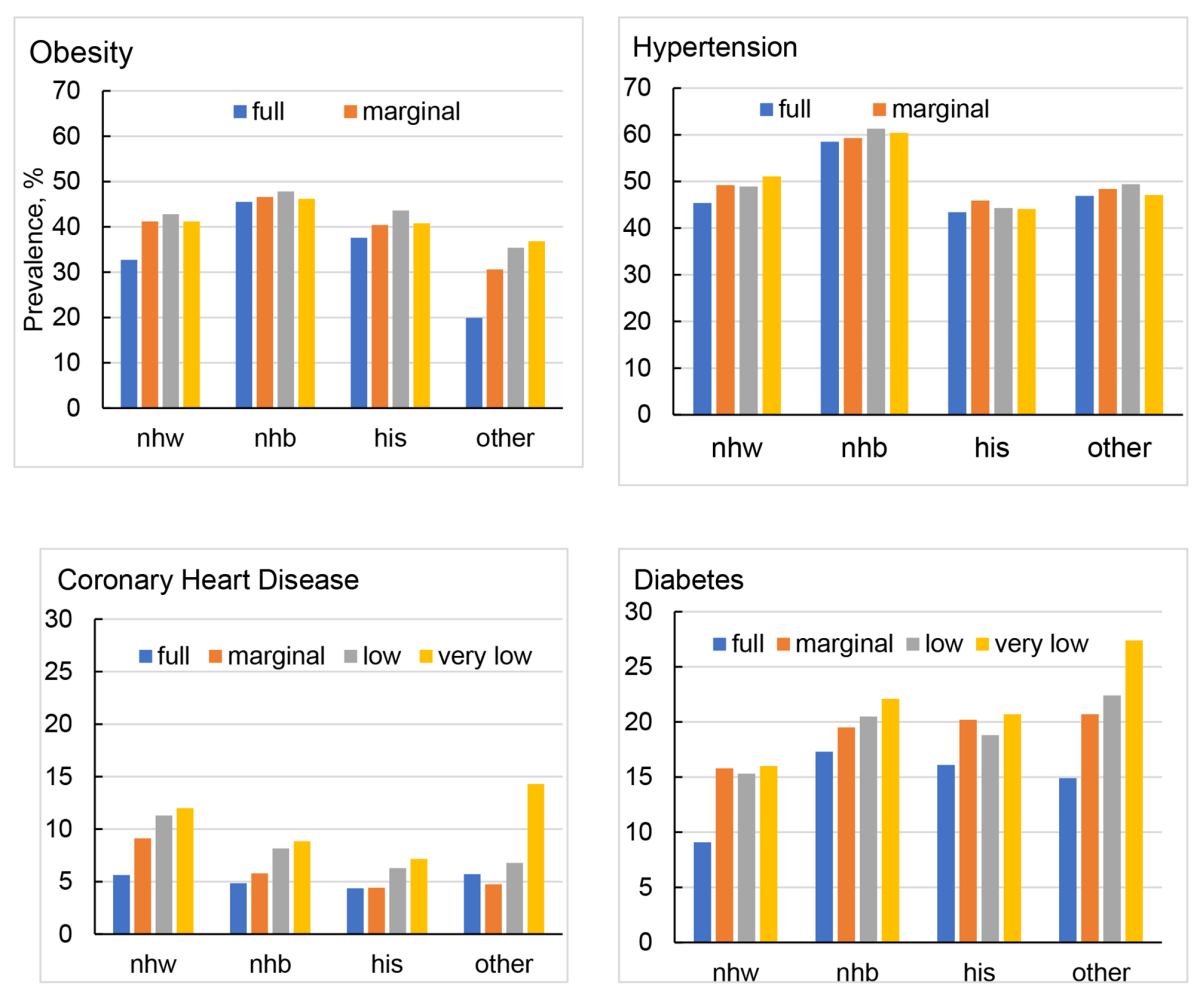
Age-adjusted prevalence of obesity, hypertension, coronary heart disease, and diabetes by food security status across race/ethnicities among US adults aged 20 years or older, NHANES 1999–2018. Abbreviations: nhw, non-Hispanic whites; nhb, non-Hispanic blacks; his, Hispanic; other, including mixed races

Figure 2 presents the disparities in age-adjusted prevalence of CMDs by racial and ethnic subgroups across food security status. Significant disparities in prevalence of CMDs were observed. For non-Hispanic white adults, the age-adjusted prevalence of obesity was 32.7% (95% CI, 31.6-33.8%) for full food secure group, 41.2% (95% CI, 38.5-44.0%) for marginal food secure group, 42.8% (95% CI, 39.5-46.2%) for low food secure group, and 41.3% (95% CI, 37.3-45.3%). For non-Hispanic black adults, the age-adjusted prevalence of obesity across food security status was 45.5% (95% CI, 44.0-46.9%), 46.6% (95% CI, 43.7-49.5%), 47.8% (95% CI, 44.6-51.0%), and 46.2% (95% CI, 42.4-49.9%). For Hispanic adults, the age-adjusted prevalence of obesity across food security status was 37.6% (95% CI, 35.8-39.4%), 40.4% (95% CI, 38.0-42.8%), 43.6% (95% CI, 40.8-46.3%), and 40.8% (35.8-46.0%). For other-race adults, the age-adjusted prevalence of obesity across food security status was 19.9% (95% CI, 17.7-22.3%), 30.6% (95% CI, 24.0-38.1%), 35.4% (95% CI, 29.9-41.4%), and 36.8% (95% CI, 30.0-44.2%). Similar patterns in prevalence of hypertension, CHD, and diabetes were observed by food security status. For example, for non-Hispanic white adults, the age-adjusted prevalence of diabetes was 9.08% (95% CI, 8.60-9.59%) for full food secure group, 15.7% (95% CI, 13.8-17.9%) for marginal food secure group, 15.3% (95% CI, 13.1-17.7%) for low food secure group, and 16.0% (95% CI, 13.3-19.1%). For non-Hispanic black adults, the age-adjusted prevalence of diabetes across food security status was 17.3% (95% CI, 16.4-18.2%), 19.5% (95% CI, 17.3-21.9%), 20.5% (95% CI, 18.6-22.4%), and 22.1% (95% CI, 19.6-24.9%). For Hispanic adults, the age-adjusted prevalence of diabetes across food security status was 16.1% (95% CI, 15.0-17.4%), 20.2% (95% CI, 18.1-22.4%), 18.8% (95% CI, 17.2-20.5%), and 20.7% (17.7-24.0%). For other-race adults, the age-adjusted prevalence of diabetes across food security status was 14.9% (95% CI, 13.3-16.7%), 20.7% (95% CI, 15.8-26.6%), 22.4% (95% CI, 19.3-25.9%), and 27.4% (95% CI, 21.5-34.3%).

## Discussion

From 1999 to 2018, the age-adjusted prevalence of CMDs were lower among participants with full food secure status compared with those with low food secure status. In general, the trend in age-adjusted prevalence of obesity and diabetes increases over time whereas trend in age-adjusted prevalence of hypertension and CHD decreases over time. However, the patterns were heterogenous by food security status. The age-adjusted prevalence of obesity increases across all food secure groups. The decreasing trend in age-adjusted prevalence of hypertension was only observed among full or marginal food secure groups but remained stable in the low and very low food security groups. The decreasing trend in age-adjusted prevalence of CHD was only observed among full food secure group but also remained stable in the low and very low food security groups. The increasing pattern of the prevalence of diabetes increases across full, marginal and low food secure groups but the prevalence of diabetes remained stable in the very low food secure group. Racial differences in prevalence of CMDs by food security status were observed. Across all race/ethnicity group, participants with full food secure have lower prevalence of CMDs compared with those with low food secure status. Non-Hispanic black adults have relatively higher prevalence of obesity, hypertension, and diabetes as compared with other race/ethnicity group. Even though our study could not provide causal explanations for such varied patterns due to the cross-sectional design, these results suggest that as a factor independent of sociodemographic characteristics, food security is significantly associated with the trend and prevalence of CMDs at the population level, with people who are food insecure more likely to develop chronic CMDs and less likely to manage their conditions.

Results from our analysis by race/ethnicity showed that, among people who were in the low and very low food security groups, both non-Hispanic Whites and minorities had a high prevalence of CMDs. Among people who were in the same food security group, there were significant racial and ethnic differences in the prevalence of CMDs with heterogenous patterns. For example, non-Hispanic black adults have higher prevalence of hypertension compared to other race/ethnicities across food security levels. Non-Hispanic white adults have relatively higher prevalence of coronary heart disease. For the full food security group, non-Hispanic black adults have higher prevalence of diabetes whereas for the low and very low food secure groups, other race/ethnicity have higher prevalence of diabetes. Those heterogenous racial patterns might be explained by the underlying social and lifestyle risk factors for each of cardiometabolic outcomes [21, 22]. Efforts must be made to address systemic racism, which affects a broad range of social determinants of health like education and income that not only contribute to disproportionate rates of food insecurity but also health disparities among racial and ethnic minorities. Additional efforts need to address other important risk factors such as poor diet that associates with a substantial proportion of deaths from CMDs [23].

Our findings are consistent with other recent studies that assessed the relationship between food security status and cardiovascular risks [24–26]. However, our study is the first one that assessed the relationship between food security and the trend of CMDs. Our results demonstrate an opportunity for federal, state, and local officials to reduce the burden of CMDs through appropriately addressing the issue of food insecurity. In addition, a recent study found that people who were food insecure were responsible for an additional $77.5 billion in health care expenditures annually compared to those who were food secure [27]. This evidence should further incentivize stakeholders to take immediate action against food insecurity because of the potential savings to the health care system and society. Effective ways to reduce food insecurity may include not only expanding existing government programs such as the Supplemental Nutrition Assistance Program (SNAP) and Women, Infants, and Children (WIC), [28] but also supporting enrollment in marginalized communities to these services by funding community-based organizations; increasing acceptance of SNAP and WIC at online food retailers; and partnering with local communities to gain trust and improve food systems. In the era of COVID-19 where food insecurity has greatly increased, these needs are only magnified. While the longer-term impact of the COVID-19 pandemic on future cardiometabolic health is yet to be determined, our results point to the critical importance of focusing on COVID-19 pandemic-related food insecurity in the short term to prevent CMD in the longer term.

This study has several limitations. First, due to small sample size, we had to combine data for every two NHANES cycles to present the trend of CMDs by food security status. This hindered us from capturing precise variations in the prevalence over years and differences in prevalence of CMDs by population subgroups. Moreover, we were unable to present trend results for Asian American populations, for which detailed race/ethnicity data became available only starting in 2011. This is problematic given the increasing and high prevalence of obesity and diabetes in Asian Americans that has been observed in national and local data [29–32]. Furthermore, several of the CMD outcomes were assessed based on self-reported information. Although self-reported data are less reliable than clinical diagnoses, previous studies showed that it was valid to use self-reported data in the NAHNES data to assess disease prevalence [33]. Additionally, findings not corrected for multiple testing should be interpreted cautiously. Finally, we used multi-year cross-sectional data, which restricted us from assessing causal relationship between food insecurity and CMDs. Large longitudinal data should be used to fill this research gap in future studies.

## Conclusions

This study found substantial and increasing disparities in CMD by food security in the U.S. In addition, food security disproportionately affected racial and ethnic minorities. These findings justify the needs for more effective public health and policy efforts to reduce food insecurity, particularly among racial and ethnic minorities. These needs have become more urgent during and after the pandemic, as improved food security would help reduce the health and economic burden of CMD and promote health equity in the long term.

## Data Availability

The dataset used in this study is publicly available and deidentified.
